# Management of Pediatric Supracondylar Humerus Fractures Using Lateral Cross-Wiring Technique Under Fluoroscopic Guidance

**DOI:** 10.7759/cureus.59029

**Published:** 2024-04-25

**Authors:** Man D Phan, Nam Q Vo, Tuong T Mai, Quoc H Truong, Khang T Truong, Phi D Nguyen

**Affiliations:** 1 Orthopaedic Surgery, Pham Ngoc Thach University of Medicine, Ho Chi Minh City, VNM; 2 Pediatric Orthopaedics, Hospital for Traumatology and Orthopaedics, Ho Chi Minh City, VNM; 3 Microsurgery and Limb Reconstruction Department, Hospital for Traumatology and Orthopaedics, Ho Chi Minh City, VNM; 4 Orthopaedic Burn Plastic Surgery Department, City Children's Hospital, Ho Chi Minh City, VNM

**Keywords:** upper extremity trauma, pediatric fractures, ulnar nerve injury, lateral cross-wiring technique, supracondylar fracture

## Abstract

Background: Supracondylar humeral fractures are the most prevalent elbow fractures in pediatric patients. Current treatment modalities typically involve closed reduction and fixation using percutaneous Kirschner wires. The lateral cross-wiring technique has demonstrated favorable functional and cosmetic outcomes, thereby reducing the incidence of ulnar nerve injury.

Objectives: This study aimed to assess the efficacy of the lateral cross-wiring technique in achieving optimal functional and cosmetic recovery while mitigating the risk of ulnar nerve injury in pediatric patients with displaced supracondylar humeral fractures.

Materials and methods: A prospective analysis was conducted on 48 patients who underwent lateral cross-wiring for displaced supracondylar fractures (Gartland type III, including extension and flexion types) of the humerus. Follow-up assessments were performed over a minimum period of eight months post-surgery.

Results: Among the 48 patients, all demonstrated satisfactory restoration of the carrying angle and functional ability postoperatively. Notably, no iatrogenic cases of radial or ulnar nerve injury were observed during the follow-up period.

Conclusion: The lateral cross-wiring technique emerges as an effective treatment option for displaced supracondylar fractures of the humerus in pediatric patients. It facilitates both functional and cosmetic recovery while concurrently reducing the risk of ulnar nerve injury, thus warranting consideration in the management of such fractures.

## Introduction

Fractures of the supracondylar region of the humerus are a common occurrence in pediatric populations, constituting approximately 70% of traumatic bone fractures in the elbow region [1-3]. These fractures are classified according to the modified Gartland classification by Wilkins, primarily based on the degree of displacement of the distal fragment, with type III fractures being the most widely addressed in contemporary practice [4,5]. Type I fractures, characterized by minimal or no displacement (less than 2mm), are typically managed conservatively with casting. Type II fractures, exhibiting displacement greater than 2mm but with intact posterior periosteum, are commonly treated with closed reduction and percutaneous Kirschner wire (K-wire) fixation, with or without cross-pinning. Type III fractures, involving complete displacement with no intact periosteum and instability, necessitate closed reduction and K-wire fixation [5]. Closed reduction and percutaneous K-wire fixation under fluoroscopic guidance have emerged as an effective treatment modality for displaced supracondylar humerus fractures in pediatric patients [2,6,7]. However, the cross-pinning technique from both condyles, while effective, presents inherent risks of ulnar nerve injury due to wire penetration. Ulnar nerve injuries associated with cross-pinning occur at rates ranging from 2% to 8% [8-10]. To mitigate the risk of ulnar nerve injury associated with cross-pinning, our research group has implemented the lateral crossed percutaneous pinning technique under fluoroscopic guidance (Dorgan's technique), altering the wire placement while maintaining the crisscross configuration. This technique aims to restore the functional and cosmetic aspects of the injured elbow while reducing the risk of ulnar nerve injury.

## Materials and methods

Study population

Inclusion Criteria

Pediatric patients diagnosed with type III supracondylar humerus fractures according to the Gartland classification, either extension or flexion type, who underwent treatment with the lateral crossed percutaneous pinning technique under fluoroscopic guidance at the Hospital for Traumatology and Orthopaedics in Ho Chi Minh City.

Exclusion Criteria

Patients with preoperative vascular or nerve injuries, fractures older than 14 days, pathological fractures, or a history of prior elbow injuries will be excluded to avoid confounding factors in the study sample.

All patients diagnosed with type III supracondylar fractures were evaluated for neurological function by both an orthopedic outpatient physician and a surgeon. Neurological examination included sensory and motor assessments of the fingers (by checking the ability to flex, extend or adduct the fingers). All type III fractures were surgically treated within 12 hours of admission.

Study methodology

The study design was a prospective descriptive study. The sample size was 48 cases of supracondylar humerus fractures treated with the lateral crossed percutaneous pinning technique. Hospital for Traumatology and Orthopaedics Ethics Committee issued approval 034625.

Data Collection Method

All eligible patients were enrolled, and data were collected using standardized data collection forms. Patients underwent clinical and radiological examinations, including anteroposterior and lateral elbow X-rays. Preoperative and postoperative clinical examinations were conducted to detect complications. Kirschner wires were removed after four weeks. Follow-up appointments were scheduled at one month, three months, and six months postoperatively.

Study Protocol

**Figure 1:**
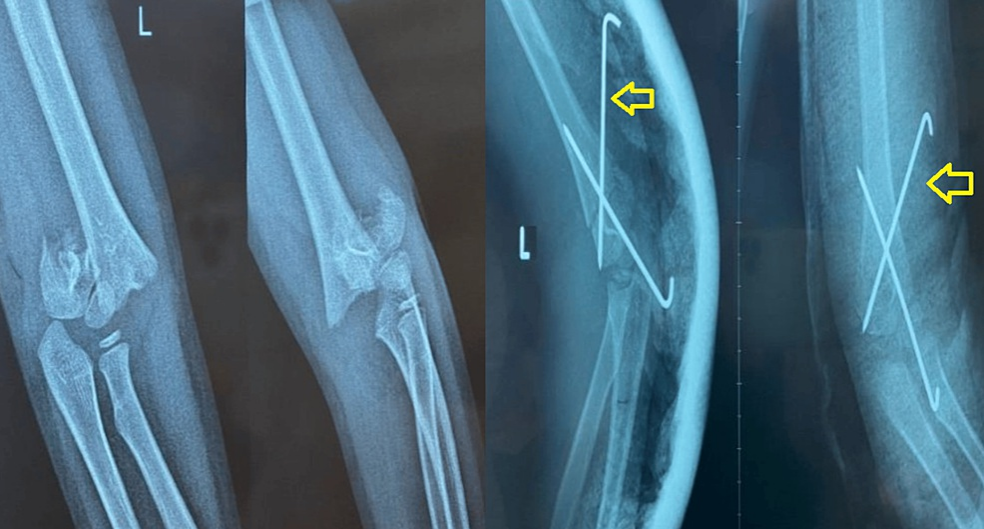
Illustrates the treatment of a supracondylar fracture utilizing lateral percutaneous pinning.

The closed reduction and percutaneous pinning technique were performed using Kirschner wires with diameters ranging from 1.6 to 1.8 mm. The pinning technique was performed under fluoroscopic guidance. The first Kirschner wire was inserted from the lateral condyle through the fracture site, traversing the cortex of the humerus. The second wire was inserted from the proximal side of the fracture, across the fracture site to the medial humeral condyle, stopping at the medial condyle cartilage (Figure 1).

The positioning of the two crossed wires was verified under fluoroscopy to ensure stability. After confirming the reduction and stability of the fracture through fluoroscopy, the Kirschner wires were bent 90 degrees outside the skin and cut to leave approximately 1-2 cm outside. Postoperatively, a splint was applied with the elbow flexed at 60-90 degrees. Neurological and vascular examinations were performed on the day after surgery, and X-rays were taken to assess the reduction. Patients were discharged and scheduled for biweekly follow-up visits. Any neurological abnormalities were documented before and after surgery through clinical examinations. Kirschner wires were removed after four weeks but in case of pin infection, we will remove the Kirshner wires at three weeks. Patients with any signs of nerve impairment were monitored biweekly to monthly until complete neurological recovery was achieved. The splint was removed after four weeks. Follow-up assessments were conducted at one month, three months, and six months post Kirschner wire removal to evaluate functional and cosmetic recovery of the injured elbow, including range of motion, clinical carrying angle according to Flynn's classification [2], and comparison with the contralateral arm. Complications such as nerve injury and infection were monitored. All patients were followed up for an average of eight months (range: 5-15 months).

## Results

All 48 patients (with characteristics in Table 1) underwent surgery utilizing the closed reduction and percutaneous pinning technique under fluoroscopic guidance at the Hospital for Traumatology and Orthopaedics. Surgery was performed within 12 hours of admission. No intraoperative complications were recorded. The mean follow-up time for all patients was 8 +/- 2.7 months.

**Table 1:** Characteristics of the Study Sample

Variables	Characteristics	Results
Age (years)	2-14	7.79 +/- 3
Gender n (%)	Male	28 (58.3)
	Female	20 (41.7)
Fracture Type n (%)	Gartland IIIA	39 (86.7)
	Gartland IIIB	6 (13.3)
Fracture Pattern n (%)	Extension	45 (93.75)
	Flexion	3 (6.25)

The mean age was 7.79 years (+/- 3). The majority of fractures were Gartland type IIIA (86.7%). There were three cases of flexion-type fractures, accounting for 6.25% of the total.

Functional outcomes assessed using Flynn's criteria [2] showed 87.5% excellent, 10.5% good, 2% fair, and 0% poor results in terms of range of motion. Cosmetic outcomes assessed through changes in the carrying angle demonstrated 75% excellent, 19% good, 2% fair, and 4% poor results. Overall, the final outcome was considered for the combined assessment, meaning if functional outcomes were excellent but cosmetic outcomes were fair, the overall result would be fair. The overall study results were 96% acceptable and 4% poor, with two cases exhibiting elbow deformity (Table 2).

**Table 2:** Functional and Aesthetic Outcomes According to Flynn Flynn Criteria [2]

Index	Degree	Outcome	Percentage (%)
Range of Motion (Function)	Excellent (0-5 degrees)	42 (87.5)	Acceptable
	Good (6-10 degrees)	5 (10.5)	
	Fair (11-15 degrees)	1 (2)	
	Poor (>15 degrees)	0 (0)	Poor
Clinical Angle Change (Aesthetic)	Excellent (0-5 degrees)	36 (75)	Acceptable
	Good (6-10 degrees)	9 (19)	
	Fair (11-15 degrees)	1 (2)	
	Poor (>15 degrees)	2 (4)	Poor

**Figure 2:**
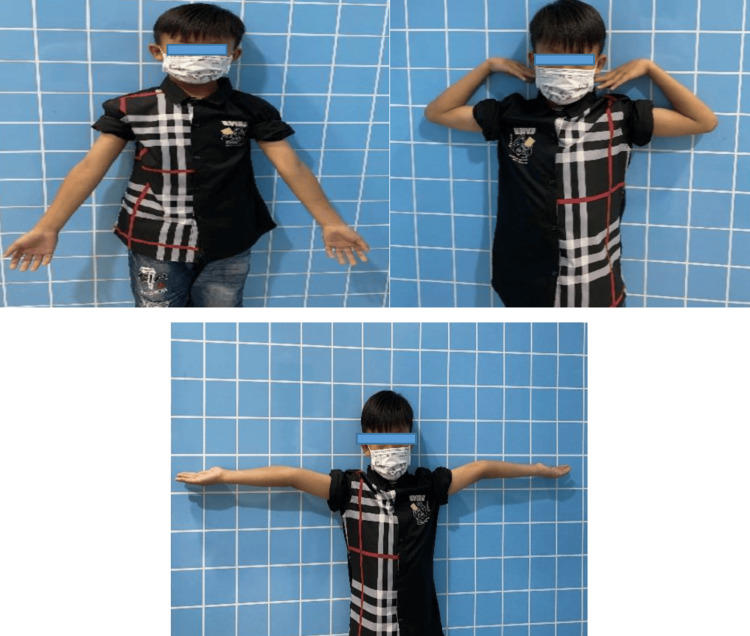
Functional and Aesthetic Recovery Outcomes of the Patient after Bone Healing.

Functional recovery of the injured elbow achieved a 100% acceptance rate (Figure 2) and 0% poor outcomes. Aesthetic function achieved a 96% acceptance rate and 4% poor outcomes.

Regarding complications (Table 3), there were no reported cases of nerve palsy, rotational malalignment, or neurovascular compromise. However, 10% of patients experienced pin site infection complications. No cases of nerve or vascular injury were observed. The most common complication was pin site infection, occurring in 10% of cases.

**Table 3:** Complication Outcomes in the Study Cohort

Complication	Number (%)
Radial Nerve	0 (0)
Ulnar Nerve	0 (0)
Medial Nerve	0 (0)
Vascular Compromise	0 (0)
Infection	5 (10)

## Discussion

Closed reduction and percutaneous cross-pinning represent the optimal configuration ensuring stability in pediatric supracondylar fractures under fluoroscopic guidance [2,9]. Ziont et al. demonstrated significantly enhanced stability with cross-pinning configuration, whereas parallel pinning was considered inferior [11]. Lee et al., utilizing a sawbone model, found that the cross-pinning model exhibited greater stability compared to the parallel pinning model in axial rotational testing [12]. The cross-pinning configuration provides better stability post-reduction, particularly in resisting rotational displacement, thus facilitating favorable bone healing outcomes. Technically, the lateral cross-pinning configuration mirrors the cross-pinning configuration. The lateral cross-pinning method merely alters the entry point of the pins while maintaining the cross-configuration.

Functional and aesthetic recovery, as per Flynn's criteria [2], demonstrated a 96% acceptance rate and 4% poor outcomes, indicating a high level of functional and aesthetic restoration. These findings align with similar studies by Shannon et al. [13] and Queally et al. [14].

Several studies employing cross-pinning techniques through both lateral and medial sides have shown rates of neurovascular compromise ranging from 2-8% [8-10,15]. Lyons et al. reported cases of neurovascular compromise resulting from cross-pinning through both sides, with pins penetrating the medial condylar leading to ulnar nerve injury, necessitating pin removal if neurovascular compromise is detected [16]. Shannon and Queally demonstrated that closed reduction and percutaneous pinning under fluoroscopic guidance using a lateral cross-pinning technique did not result in neurovascular compromise in their study cohorts [13,14]. Similarly, in all 48 patients, no cases of neurovascular compromise were noted because the pin positions were adjusted to avoid neurovascular structures. Theoretically, the radial nerve may be at risk during the pinning process at the distal third of the humerus; however, at this location, the nerve lies anteriorly [17]. Bloom et al. suggested that pins placed in the distal end of the humerus from anterior to posterior may pose a risk to the radial nerve; therefore, to avoid this, pin entry points should be from the midshaft of the humerus posteriorly, with pin direction from posterior to anterior [18].

The most common complication encountered is superficial pin site infection (10%), which is higher compared to Shannon et al. (5%) and Queally et al. (7%) [13,14]. These infections are mild and non-articular and can be managed with oral antibiotics and wound care. They represent a minor complication that resolves completely upon pin removal.

This study is constrained by several limitations. Primarily, it is characterized as a case series, lacking a control group for comparative analysis against alternative methodologies. Furthermore, the research was conducted exclusively within a single center, thereby potentially limiting the generalizability of its findings. Subsequent investigations involving larger cohorts and multi-center collaborations are imperative to substantiate the efficacy of the surgical approach under scrutiny.

## Conclusions

In summary, the treatment of supracondylar fractures using the lateral cross-pinning technique under fluoroscopic guidance across 48 cases presents an additional option in the repertoire of fracture management techniques. This technique yields favorable outcomes in both functional and aesthetic recovery. Furthermore, the lateral cross-pinning method under fluoroscopic guidance offers the advantage of minimizing the risk of ulnar nerve compromise.
